# SDCCAG8 Interacts with RAB Effector Proteins RABEP2 and ERC1 and Is Required for Hedgehog Signaling

**DOI:** 10.1371/journal.pone.0156081

**Published:** 2016-05-25

**Authors:** Rannar Airik, Markus Schueler, Merlin Airik, Jang Cho, Kelsey A. Ulanowicz, Jonathan D. Porath, Toby W. Hurd, Simon Bekker-Jensen, Jacob M. Schrøder, Jens S. Andersen, Friedhelm Hildebrandt

**Affiliations:** 1 Department of Medicine, Division of Nephrology, Boston Children’s Hospital, Boston, Massachusetts, United States of America; 2 Department of Pediatrics, Division of Nephrology, Children’s Hospital of Pittsburgh of UPMC, Pittsburgh, Pennsylvania, United States of America; 3 Medical Research Council Human Genetics Unit, Institute of Genetics and Molecular Medicine, University of Edinburgh, Edinburgh, United Kingdom; 4 NNF Center for Protein Research, University of Copenhagen, Faculty of Health Sciences, Copenhagen, Denmark; 5 Department of Biochemistry and Molecular Biology; University of Southern Denmark, Odense M, Denmark; 6 Howard Hughes Medical Institute, Chevy Chase, Maryland, United States of America; University of Illinois at Chicago, UNITED STATES

## Abstract

Recessive mutations in the *SDCCAG8* gene cause a nephronophthisis-related ciliopathy with Bardet-Biedl syndrome-like features in humans. Our previous characterization of the orthologous *Sdccag8*^*gt/gt*^ mouse model recapitulated the retinal-renal disease phenotypes and identified impaired DNA damage response signaling as an underlying disease mechanism in the kidney. However, several other phenotypic and mechanistic features of *Sdccag8*^*gt/gt*^ mice remained unexplored. Here we show that *Sdccag8*^*gt/gt*^ mice exhibit developmental and structural abnormalities of the skeleton and limbs, suggesting impaired Hedgehog (Hh) signaling. Indeed, cell culture studies demonstrate the requirement of SDCCAG8 for ciliogenesis and Hh signaling. Using an affinity proteomics approach, we demonstrate that SDCCAG8 interacts with proteins of the centriolar satellites (OFD1, AZI1), of the endosomal sorting complex (RABEP2, ERC1), and with non-muscle myosin motor proteins (MYH9, MYH10, MYH14) at the centrosome. Furthermore, we show that RABEP2 localization at the centrosome is regulated by SDCCAG8. siRNA mediated RABEP2 knockdown in hTERT-RPE1 cells leads to defective ciliogenesis, indicating a critical role for RABEP2 in this process. Together, this study identifies several centrosome-associated proteins as novel SDCCAG8 interaction partners, and provides new insights into the function of SDCCAG8 at this structure.

## Introduction

Mutations in *SDCCAG8* cause a nephronophthisis-related ciliopathy with multiple organ involvement, including retinal degeneration, cognitive defects, renal failure, hypogonadism, obesity and infrequently clinodactyly [1, 2]. We recently recapitulated several of these human disease phenotypes in a mouse model of *Sdccag8*, demonstrating both cilia dependent and independent functions of Sdccag8 [3, 4]. Abnormalities of the cilium are critical for features that are dependent on signaling pathways that require intact cilia for their function, such as the hedgehog (Hh) signaling pathway in mammals [5]. Since the demonstration that mammalian Hh signal reception and initial processing take place within the primary cilium, this microtubule-based structure became central in understanding the disease mechanisms of a wide spectrum of diseases, so called ciliopathies [5, 6].

Primary cilia assembly is a process in which ciliary components are transported from the Golgi to the ciliary base near the basal body, where they interact with large multi-subunit intraflagellar transport apparatus (IFT) and BBSome protein complexes that transport the material into cilia [7, 8]. Recent work has suggested that recruiting and loading of membrane proteins to the BBSome complex takes place at the centriolar satellites, as deficiency in centriolar satellite proteins CEP290, BBS4, OFD1 and CEP131/AZI1 affects ciliogenesis [9–13]. Intracellular transport of the Golgi-derived ciliary cargo is performed by vesicular transport components, small GTPases and their effector molecules, including Rab11, Rab8, Rabin8 and RABEP1/Rabaptin-5 proteins [8, 14–17]. Besides RAB8 and RAB11, also RAB6 was recently implicated in membrane trafficking by demonstrating its involvement in polycystin-1 transport to the primary cilium [18].

Here we show that mice deficient in *Sdccag8* in addition to the retinal-renal phenotype, have developmental abnormalities of the skeleton and limbs consistent with disruption of hedgehog signaling. By cell culture analysis we demonstrate impaired ciliogenesis and reduced responsiveness to a hedgehog signaling activator, SAG, in *Sdccag8*^*gt/gt*^ derived mouse embryonic fibroblasts. To further investigate the function of SDCCAG8 and to define the SDCCAG8 protein interaction network at the centrosome we performed a SILAC-assay [19]. Besides determining the composition of the SDCCAG8 complex at the centrosome we uncovered several hitherto unknown centriolar proteins. We demonstrate that the localization of the newly identified SDCCAG8 interacting protein RAB GTPase binding effector protein 2 (RABEP2) is regulated by SDCCAG8, and that RABEP2 is a critical regulator of ciliogenesis in hTERT-RPE1 cells. Together, these findings reveal new insights into the function of SDCCAG8 at the centrosome.

## Materials and Methods

### Mouse Breeding and Maintenance

The experimental protocol was reviewed and approved by the Animal Care Committee of the Boston Children’s Hospital. Generation of *Sdccag8*^*gt/gt*^ mice has been described previously [3]. *Sdccag8* wild type or heterozygous littermates were used as controls for mutant mice. For timed matings; noon on the day a plug was found was designated as embryonic day 0.5 (E0.5).

### Skeletal preparation

Alcian blue and alizarin red staining was done using standard protocols. Briefly, hind limbs were dissected, fixed in 95% ethanol for 2 days, kept in acetone for 2 days and rinsed with water. Staining cocktail (1 volume 0.3% alcian blue in 70% EtOH, 1 volume 0.1% alizarin red in 95% EtOH, 1 volume 100% acetic acid and 17 volume 100% EtOH) was added and bones incubated at RT for 5–10 days until visible through surrounding tissue and fully stained. Surrounding tissue was cleared by immersion in 1% KOH for 24 h followed by a graded 1% KOH/glycerol series. Stained skeletal preparations were stored and photographed in 80% glycerol.

### Generation of Mouse Embryonic Fibroblasts

Mouse embryonic fibroblasts (MEF) were established from wild type and *Sdccag8*^*gt/gt*^ E13.5 embryos and cultured in DMEM with 10% FBS and penicillin/streptomycin.

### Plasmid cloning

To generate GFP-RABEP2-PACT centrosomal targeting construct, full-length RABEP2 coding region (Accession:BC058900, Clone ID:5415624, Dharmacon) was cloned in the pEGFP-C1-PACT plasmid, a gift from A.Kraemer [20].

### Immunofluorescence Analysis

E10.5 embryos were fixed in 4% (w/v) paraformaldehyde (PFA) in PBS at 4°C. Embryos were then immersed in 15% and 30% sucrose and embedded in Tissue Freezing Medium (Triangle Biomedical Sciences, Inc.). Sections were taken at 8 μm. For immunostaining sections were blocked in 10% donkey serum/1% BSA and permeabilized in 0.1% Tween-20.

Co-localization coefficients between γ-tubulin, acetylated α-tubulin or polyglutamylated tubulin and ERC1, RABEP2 or CEP131 at centrosomes (30 centrosomes analyzed per sample) were determined using Fiji JACoP colocalization coefficient software [21]. Using this software, Manders overlap coefficient scores can range from 0 to 1 and represent 0 to 100% co-localization within a given region, respectively. Centrosomal localization of ERC1, RABEP2 and CEP131 was quantitated by measuring the number of positive pixels of the respective proteins in acetylated α-tubulin or polyglutamylated tubulin positive centrosomes, using Fiji software [21].

### Antibodies

Primary antibodies used were as follows: rabbit polyclonal anti-CEP164 was a gift from Erich Nigg (University of Basel, Switzerland), rabbit polyclonal anti-ERC1 (A302-698A, Bethyl Laboratories), rabbit polyclonal anti-CEP131 (ab99379, Abcam), rabbit polyclonal anti-RABEP2 (ab138279, Abcam), mouse monoclonal anti-SDCCAG8 (ab67098, Abcam), rabbit anti-DYKDDDDK Tag (#2368, Cell Signaling), mouse monoclonal anti-γ-tubulin (GTU-88, Sigma), rabbit anti-acetylated α-tubulin (#5335, Cell Signaling), mouse monoclonal anti-polyglutamylated tubulin (T9822, Sigma-Aldrich), rabbit polyclonal anti-CCDC41 (CEP83) (HPA038161, Atlas), rabbit polyclonal anti-FBF1 (HPA023677, Atlas), chicken anti-GFP (GFP-1020, Aves). Antibodies against FoxA2 (clone 4C7), Hb9 (clone 81.5C10), Nkx2.2 (clone 74.5A5) and Nkx6.1 (clone F65A2) are from the Developmental Studies Hybridoma Bank. Secondary antibodies were: donkey anti-mouse Alexa Fluor 488, donkey anti-mouse Alexa Fluor 594, donkey anti-rabbit Alexa Fluor 488 and donkey anti-rabbit Alexa Fluor 594 (Molecular Probes), goat anti-chicken Alexa Fluor 488 (Molecular Probes). Samples were mounted in ProlongGold (Molecular Probes) and images captured on a Leica TSC 5SP X confocal microscope (Leica Microsystems).

### siRNA Knockdown Assay

hTERT-RPE1 cells were cultured in DMEM in 10% FBS. 25,000 cells were seeded per well in 24-well dishes (plastic and glass bottom) in triplicate 24 h before being transfected with 50 nM Dharmacon ON-TARGETplus siRNA against human SDCCAG8 (smartpool, L-045871-01-0005), human RABEP2 (smartpool, L-009001-01-0005), human CEP131 (smartpool, L-023335-00-0005) or control (NS; D-001810-10-20) oligos.

### RNA Extraction and qRT–PCR

RNA was isolated from wild type, *Sdccag8*^*wt/gt*^ and *Sdccag8*^*gt/gt*^ MEF cells and adult kidney cells using RNeasy Mini Kit (Qiagen), and reverse transcription was performed using Superscript III (Invitrogen). Quantitative real-time PCR was carried out using Sybr Green (Qiagen) and run on a MyiQ Single-Color Real-Time PCR Detection System (Bio-Rad Laboratories, Inc). Data were normalized to *Gapdh*. Primer sequences are available upon request.

### SDCCAG8 co-immunoprecipitation assay

HEK293T cells were transfected with a C-terminal FLAG-tag expression vector with or without human full-length and truncated SDCCAG8 cDNAs. Two days after transfection, the cell lysates were prepared using IP lysis buffer (Pierce). Cleared cell lysates were produced by centrifugation of the resulting samples at 16,000 × g for 30 min at 4°C, and subjected to immunoprecipitation using anti-Flag M2 beads (Sigma). Gel electrophoresis of immunoprecipitation eluents was performed using the NuPAGE system (Invitrogen). Samples were resolved on 4–12% Bis-Tris gels in MOPS buffer, transferred to a nitrocellulose membrane which was then probed for the protein of interest using antibodies diluted in TBS containing 5% milk and 0.1% Tween-20 (Sigma).

### Statistical Methods

T-test was used to compare data between two groups. Multiple comparisons involving more than three groups were analyzed by ANOVA. Significance was determined at P < 0.05 and represented by * to denote P<0.05, ** P<0.01, *** P<0.001, **** P<0.0001. Data were analyzed using Prism 6 software (GraphPad Software, San Diego, CA) and are given as the mean ± SEM.

## Results

### *Sdccag8*^*gt/gt*^ mice exhibit hind limb and rib cage malformations

**Fig 1:**
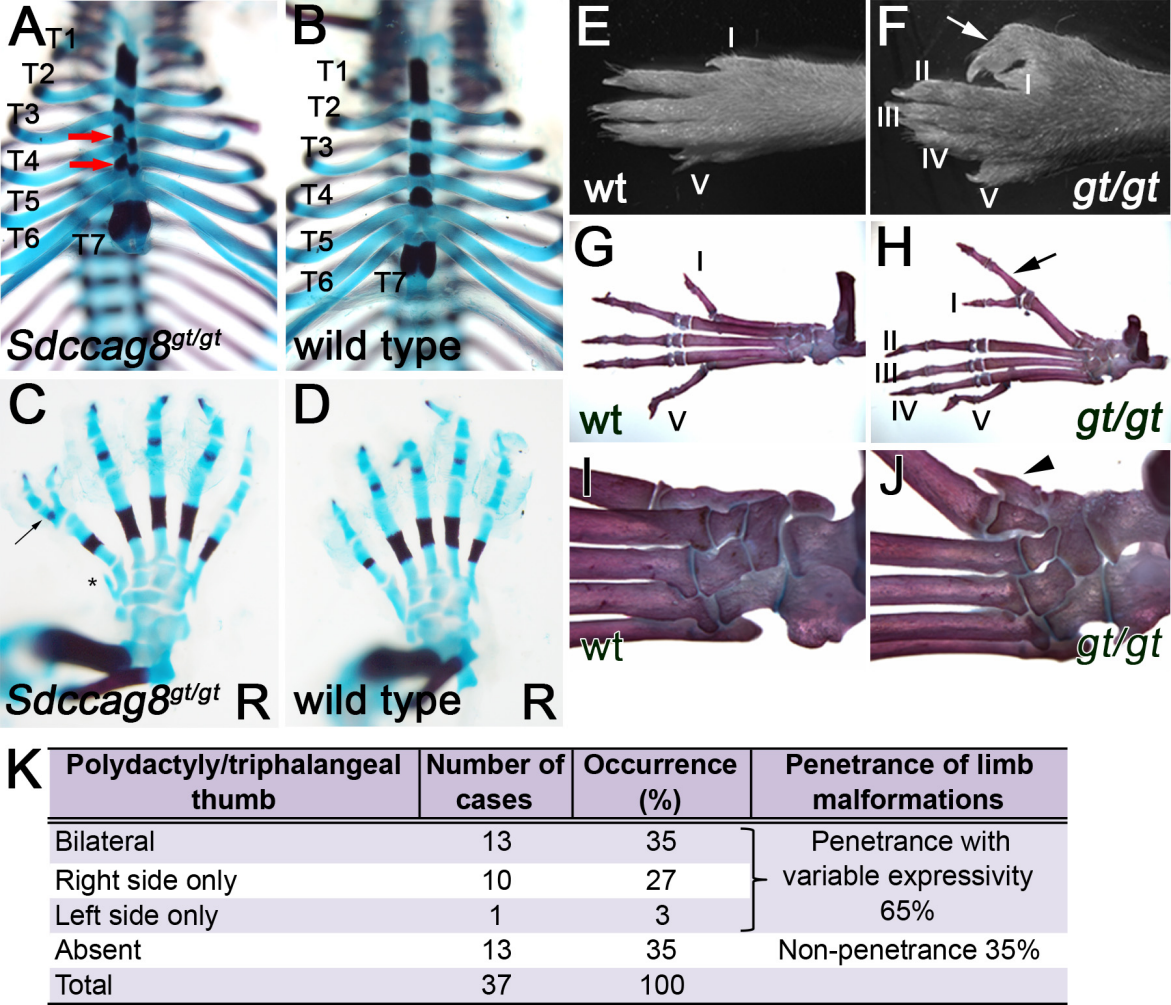
*Sdccag8*^*gt/gt*^ mice exhibit rib cage and pre-axial polydactyly phenotypes. (**A**–**D**) Skeletal preparation of E18.5 rib cage and hind limbs, demonstrate misalignment (red arrows) of sternum ossification centers in *Sdccag8*^*gt/gt*^ embryos (**A**), when compared to wild type control (**B**), and formation of triphalangeal polydactyly (arrow) in hind limbs (**C**), when compared to wild type control (**D**). Asterisk indicates a bony pre-axial polydactylous structure in **C**. Thoracic vertebral segments are identified for T1-T7, R denotes right paw. (**E**–**J**) Anatomical overview and skeletal preparations of P30 wild type (**E**,**G**,**I**) and *Sdccag8*^*gt/gt*^ (**F**,**H**,**J**) right hind limbs. Fingers are numbered with Roman numerals starting with the thumb (arrows indicate triphalangeal polydactylous pre-axial thumbs in *Sdccag8*^*gt/gt*^ hind limbs (**F** and **H**). Skeletal preparations of the limbs demonstrate forking of the *Sdccag8*^*gt/gt*^ thumb after the first phalange (**H**). An additional rudimentary finger-like bone is located pre-axial to the defective thumb (arrowhead) (**J**). *wt*, *Sdccag8* wild type allele; *gt*, *Sdccag8* gene-trap allele. (**K**) Quantitation of the hind limb polydactyly/triphalangeal thumb phenotype in *Sdccag8*^*gt/gt*^ mice reveals a variable expressivity of this feature. The most frequent phenotype is the occurrence of bilateral polydactyly/triphalangeal thumbs in *Sdccag8*^*gt/gt*^ mice at 35%. Structurally, less severe phenotypes of right side only or left side only polydactyly/triphalangeal thumbs occur in 27% and 3% of the cases, respectively.

Previously, we reported that *Sdccag8*^*gt/gt*^ mice recapitulate the human *SDCCAG8*-deficiency phenotypes of retinal and renal degeneration [1, 2]. Upon further evaluation of *Sdccag8*^*gt/gt*^ mouse phenotypes, we found that loss of SDCCAG8 function causes developmental bone malformations in the mutant mice (**Fig 1**). Specifically, skeletal preparations of *Sdccag8*^*gt/gt*^ embryos at E18.5 revealed rib cage abnormalities characterized by misalignment of the sternal ossification centers (**Fig 1A** and **1B**) and pre-axial polydactyly with triphalangeal thumbs of the hind limbs (Fig **1C** and **1D**). To analyze the structural defects of the distal limbs at postnatal stage we performed limb skeletal preparations of P30 mice (**Fig 1E**–**1J**). The analysis revealed an incomplete penetrance of the skeletal abnormalities in mutant mice, with the polydactyly occurring in 65% of homozygous *Sdccag8*^*gt/gt*^ animals and having preferentially bilateral (35%) or right side only (27%) presentation (**Fig 1K**). Besides the phenotype of triphalangeal thumbs we identified additional pre-axial bone malformations on hind limbs (**Fig 1C** and **1J**). Although polydactyly has not been reported in individuals with mutation in *SDCCAG8* they do infrequently manifest post-axial clinodactyly [1, 2].

### SDCCAG8 is required for ciliogenesis and Hh signaling

**Fig 2:**
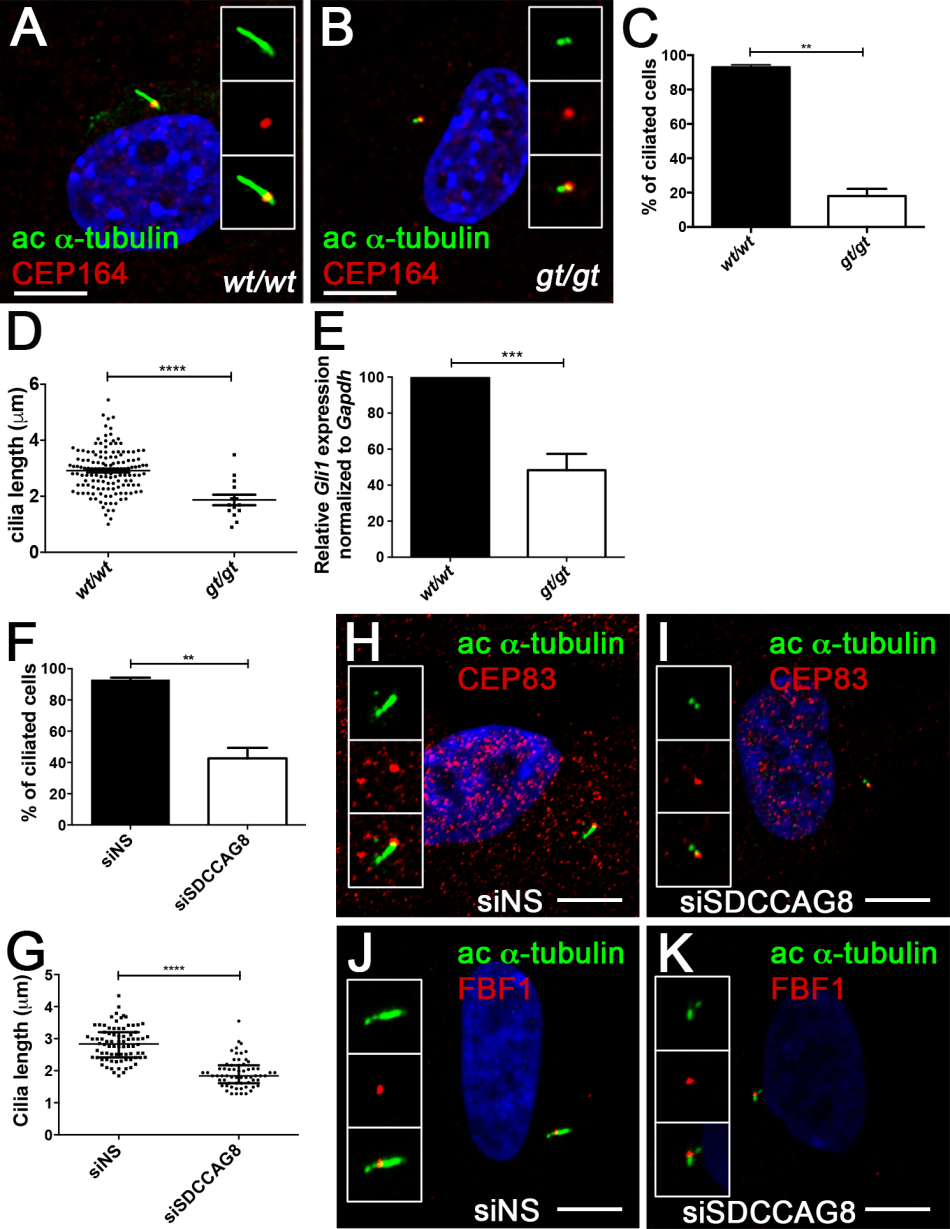
Sdccag8 is required or cilia formation and Hh signaling. (**A**,**B**) *Sdccag8*^*gt/gt*^ cells have shorter cilia. Immunofluorescence staining of cultured mouse embryonic fibroblasts derived from wild type (**A**) and *Sdccag8*^*gt/gt*^ (**B**) mice using antibodies against distal appendage marker CEP164 (red) and cilia marker acetylated tubulin (green), demonstrate shorter cilia in *Sdccag8*^*gt/gt*^ cells (**B**). (**C**) Quantitation of the percentage of ciliated cells for wild type and *Sdccag8*^*gt/gt*^ cells after serum starvation (48 hrs). Significantly less *Sdccag8*^*gt/gt*^ cells grow cilia compared to wild type cells (18% vs. 93%, n = 100 for both genotypes, **p = 0.0018). (**D**) *Sdccag8*^*gt/gt*^ MEFs have significantly shorter cilia (1.9 ± 0.2 μm, n = 20) compared to wild type cells (2.9 ± 0.0 μm, n = 142, ****p<0.0001). (**E**) *Sdccag8*^*gt/gt*^ MEFs have attenuated response to Hh signal agonist SAG. qRT-PCR analysis demonstrates reduced levels of Hh pathway target gene *Gli1* in SAG treated *Sdccag8*^*gt/gt*^ MEFs compared to wild type cells (N = 3). (**F**) Knockdown of *SDCCAG8* causes a reduction in cilia formation in hTERT-RPE1 cells. Only 43% of SDCCAG8 knockdown cells grew cilia compared to 94% wild type cells (**p = 0.0092). (**G**) Cilia length is significantly reduced in SDCCAG8 knockdown cells (1.9 ± 0.1 μ, n = 63) compared to wild type cells (2.8 ± 0.1 μ., n = 80, ****p<0.0001). Scale bar: (**A**,**B**) 7.5 μm. *wt*, *Sdccag8* wild type allele; *gt*, *Sdccag8* gene-trap allele. (**H**,**I**,**J**,**K**) Immunofluorescence staining of control siNS hTERT-RPE1 cells (**H**,**J**) and siSDCCAG8 hTERT-RPE1 cells (**I**,**K**) with antibodies against acetylated tubulin (green) and distal appendage markers CEP83 (red) (**H**,**I**) or FBF1 (red) (**J**,**K**), reveals no abnormalities in the formation of centriolar distal appendages despite the loss of cilia in *SDCCAG8* knockdown cells (**I**,**K**) compared to control cells transfected with non-specific siRNA (**F**,**H**).

Formation of pre-axial polydactyly with triphalangeal thumbs has been attributed to disruptions in the Hedgehog signaling pathway, that requires the presence of intact primary cilia for pathway activation [5, 22]. Our previous studies indicated that *Sdccag8*^*gt/gt*^ mice do not have a gross ciliogenesis defect *in vivo* in renal tubule cells [3]. We therefore asked whether there is a more subtle requirement for SDCCAG8 function in cilia formation using mouse embryonic fibroblast (MEF) cultures from E13.5 wild type and *Sdccag8*^*gt/gt*^ embryos. We first evaluated whether loss of *Sdccag8* affects ciliogenesis in MEFs. Serum starved MEFs were stained with antibodies against acetylated α-tubulin, a component of the ciliary axoneme and CEP164, a component of the transition zone (**Fig 2A and 2B**) [23]. *Sdccag8*^*gt/gt*^ MEFs largely failed to develop primary cilia, forming only a very short acetylated α-tubulin positive structure emanating from the CEP164 positive basal body (**Fig 2B**). We found that only 18% of *Sdccag8*^*gt/gt*^ MEFs were able to assemble the primary cilium, compared to 93% of wild type cells (**p = 0.0018, **Fig 2C**), demonstrating that Sdccag8 is required for primary ciliogenesis in cell culture. Furthermore, the cilia of *Sdccag8*^*gt/gt*^ cells (1.9 ± 0.2 μm), were significantly shorter that those of wild type cells (2.9 ± 0.0 μm ****p<0.0001; **Fig 2D**). To examine whether Hh signaling is impaired in Sdccag8-deficient cells, ciliated MEFs were treated for 18 hrs with a small molecule Smoothened agonist (SAG) to activate the Hh signaling pathway. Quantitative RT-PCR for *Gli1*—a direct transcriptional target of hedgehog signaling, showed a 50% decrease in Hh pathway activation in *Sdccag8*^*gt/gt*^ MEFs compared to wild type control cells (**Fig 2E**).

We next asked whether acute knockdown of *SDCCAG8*, using siRNA, as we have performed earlier causes impaired ciliogenesis in hTERT-RPE1 cells [1]. Indeed, we observed a significant decrease in the fraction of ciliated hTERT-RPE1 cells transfected with siSDCCAG8 (43 ± 7%, n = 70, triplicate experiments) compared to cells transfected with non-targeting control siNS (94 ± 2%, n = 80, p = 0.0092, triplicate experiments; **Fig 2F**). Moreover, the cilia that did form in *SDCCAG8* knockdown cells (1.9 ± 0.1 μ1, n = 63) were significantly shorter than in control cells (2.8 ± 0.1 μ, n = 80, p<0.0001; **Fig 2G**). SDCCAG8 has been previously described as a component of the subdistal appendages at the basal body, but not of the distal appendages [1]. In order, to demonstrate that loss of SDCCAG8 did not affect the integrity of the distal appendages we performed an immunofluorescence analysis against distal appendages markers–CEP83 and FBF1 (**Fig 2H**–**2K**) [24]. Indeed, CEP83 and FBF1 localization to the distal end of the mother centriole did not appear affected in *SDCCAG8* knockdown cells, despite the lack of cilia formation (**Fig 2I** and **2K**) compared to control cells (**Fig 2H** and **2J**). Together, our results indicate that SDCCAG8 is required for ciliogenesis in mouse embryonic fibroblasts and in hTERT-RPE1 cells, and that loss of *Sdccag8* abrogates Hh signaling. These findings are consistent with the polydactyly phenotype observed in *Sdccag8*^*gt/gt*^ mice.

### Sdccag8 is not required for normal dorsal–ventral patterning in the neural tube

Although, our data indicated that SDCCAG8 is critical for Hh signaling in cell culture and likely regulates Hh signaling during autopod development, we did not observe other overt Hh deficiency phenotypes in *Sdccag8*^*gt/gt*^ mice. However, it was recently shown that Sdccag8 plays a role in neuronal migration in the developing cortex, a process that involves Shh signaling [4, 25]. We thus decided to investigate whether *Sdccag8*^*gt/gt*^ mice display deficiencies in Hh signaling in the neural tube patterning which is regulated by Shh signaling. High levels of Shh are required for the most ventral cell fates, the floorplate and V3 interneurons of the neural tube, whereas lower levels specify motor neurons and interneuron fates. We used an antibody against FoxA2 to stain floor plate cells, Hb9 to stain motor neurons, Nkx2.2 to stain V3 interneuron progenitors and Nkx6.1 to stain motor neuron progenitors. Our analysis reveals that the neural tube of *Sdccag8*^*gt/gt*^ mutants is histologically indistinguishable from wild type embryos at E10.5, and the specification of their floor plate and intermediate neuronal cell populations in the neural tube is unchanged (**S1 Fig**). Specifically the expression domains of FoxA2 (**S1A and S1B Fig**), Hb9 (**S1C and S1D Fig**), Nkx2.2 (**S1E and S1F Fig)** and Nkx6.1 (**S1G and S1H Fig**) appear unaffected in *Sdccag8*^*gt/gt*^ mice, indicating that Sdccag8 function is not essential for Shh regulation in this tissue.

### SDCCAG8 and its interacting proteins form a distinct functional module at the centrosome

Recent proteomics studies have defined several protein complexes of ciliopathy proteins (BBS, NPHP, MKS, IFT) that form functional modules at the centrosome or within the cilium [26, 27]. These protein complexes are frequently involved in ciliogenesis through regulating transport of materials to the basal body and subsequently to the cilium. To shed further light on the function of SDCCAG8 within its localization at the base of the cilium and to identify proteins that interact with SDCCAG8 at the centrosome, we employed a proteomic strategy using stable isotope labeling with amino acids in cell culture (SILAC). This method was previously used to identify novel centrosomal proteins in an unbiased manner [19]. SILAC assay was performed in hTERT-RPE1 cells, which harbored a doxycycline inducible GFP-tagged full-length SDCCAG8 over-expression construct [1]. SILAC experiments were performed in duplicates, and specific interactors were identified by comparing the heavy to light ratios of precipitated proteins from cell lysates, as described before [19].

Using a 1.7 fold difference in heavy to light ratios of precipitated proteins as a cut-off from three experiments produced a final dataset of 19 proteins co-purifying with SDCCAG8 (**Table 1**). Our experiments identified two previously described centrosomal proteins–a known SDCCAG8 interacting protein, oral-facial-digital syndrome 1 protein (OFD1) [1] and a centrosomal satellite protein, centrosomal protein 131kDa (CEP131) [12] (**Table 1**). In addition, components of the endosomal vesicle trafficking complex and trans-Golgi network, such as RAB GTPase binding effector protein 2 (RABEP2), ELKS/RAB6-interacting/CAST family member 1 (ERC1), and ELKS/RAB6-interacting/CAST family member 2 (ERC2) were highly represented. Furthermore, we identified as SDCCAG8 interaction partners aminoacyl-tRNA synthetases (KARS, DARS) and a protein required for the assembly and stability of the aminoacyl-tRNA synthase complex (AIMP2). We also found that SDCCAG8 interacts with non-muscle myosin II motor proteins MYH9, MYH10 and MYH14, that associate with the actin cytoskeleton and have been shown to regulate cilia formation in various animal and cell based model systems [28, 29].

**Table 1:** Identification of human SDCCAG8 complex proteins^a^.

Gene symbol	Full name	Ratio H/L Normalized
SDCCAG8	Serologically defined colon cancer antigen 8	16.1
RABEP2	Rab GTPase-binding effector protein 2	6.4
OFD1	Oral-facial-digital syndrome 1 protein	5.7
KARS	Lysyl-tRNA synthetase	3.4
AIMP2	Aminoacyl tRNA synthetase complex-interacting multifunctional protein 2	3.1
ERC1	ELKS/RAB6-interacting/CAST family member 1	2.9
ERC2	ELKS/RAB6-interacting/CAST family member 2	2.5
MYH14	Myosin heavy chain 14	2.5
SDCBP	Syntenin-1	2.0
GYG1	Glycogenin-1	2.0
MYO1C	Myosin-Ic	1.9
SPTAN1	Spectrin alpha chain, brain	1.9
DARS	Aspartyl-tRNA synthetase, cytoplasmic	1.8
MYH9	Myosin heavy chain 9	1.8
LRRFIP2	Leucine-rich repeat flightless-interacting protein 2	1.8
CEP131	Centrosomal protein 131kDa	1.8
MYH10	Myosin heavy chain 10	1.7
PLEC1	Plectin-1	1.7
SPTBN1	Spectrin beta chain, brain 1	1.7

^a^SILAC enrichment factors (3 independent experiments) of proteins co-immunoprecipitating with GFP-tagged SDCCAG8. This list contains all 19 proteins uncovered by the SILAC assay, with a normalized ratio of heavy to light peptides >1.7.

**Fig 3:**
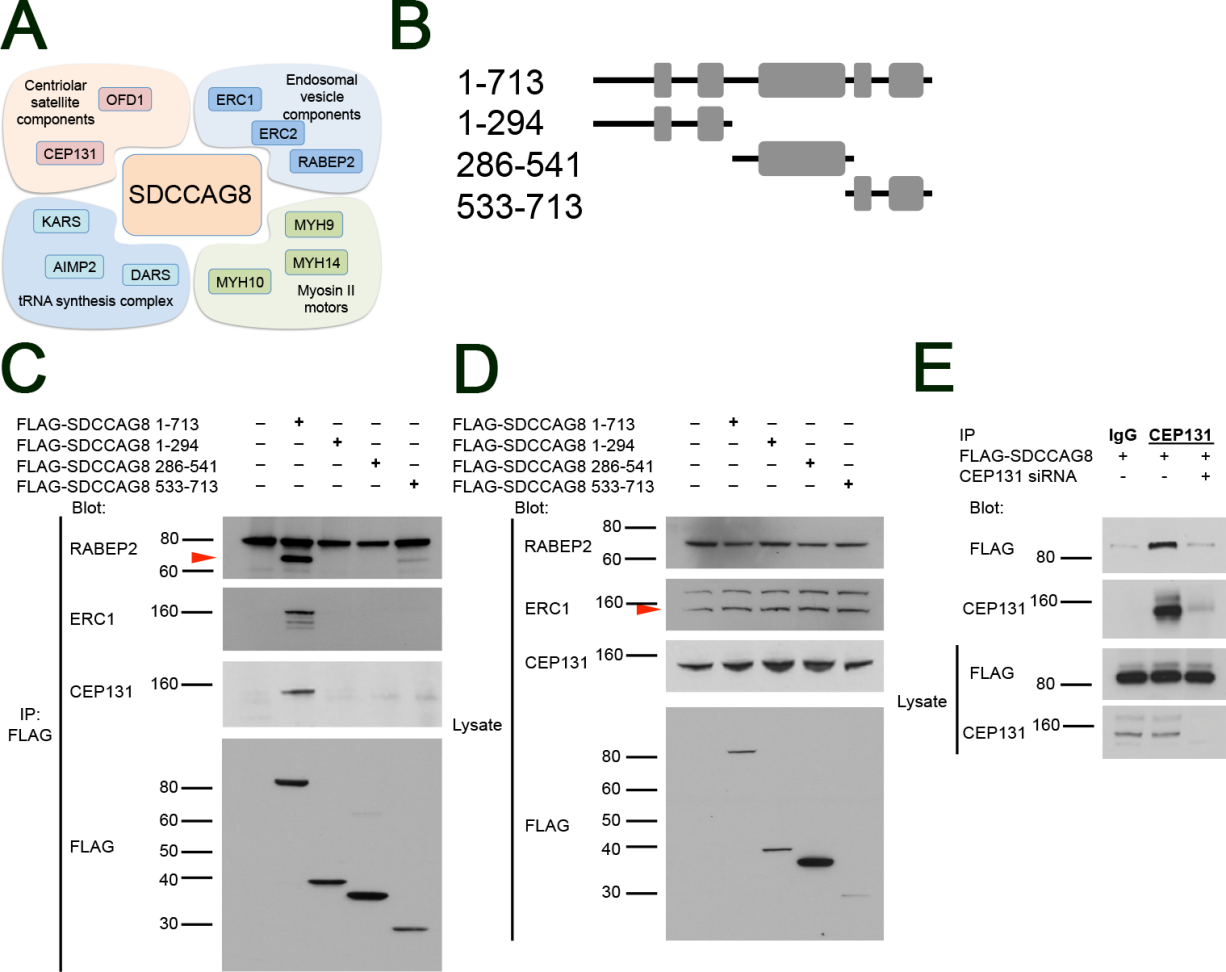
SDCCAG8 associates with a characteristic set of proteins at the centrosome. (**A**) The Sdccag8 interacting proteins discovered by SILAC as centrosomal components belong to 4 functional groups, i.e. centriolar satellite components, endosomal vesicle components, tRNA synthesis complex proteins, and myosin type II motors involved in ciliogenesis. (**B**) Overview of the SDCCAG8 constructs used in this study. Numbering corresponds to amino acid positions. Gray boxes designate coiled-coil domains. (**C**) SDCCAG8 interacts with RABEP2, ERC1, and CEP131. FLAG-tagged full-length and truncation constructs of SDCCAG8 were immunoprecipitated from extracts of HEK293 cells transfected with each construct and analyzed by Western blot. Blots were probed for FLAG, or for endogenous RABEP2, ERC1, and CEP131 respectively. Only full-length FLAG-tagged SDCCAG8, but not truncated SDCCAG8 constructs co-immunoprecipitate RABEP2, ERC1 or CEP131, except for the C-terminal SDCCAG8 fragment that weakly co-immunoprecipitated RABEP2. (**D**) Western blotting for endogenous proteins in whole cell lysates shows equal loading. (**E**) CEP131 co-immunoprecipitates FLAG-SDCCAG8. Lysates from cells transfected with FLAG-SDCCAG8 and with non-specific control siRNA or with siCEP131 were subjected to immunoprecipitation using anti-CEP131 antibodies or normal IgG. While anti-CEP131 antibodies co-immunoprecipitated FLAG-SDCCAG8 from control lysates, *CEP131* knockdown abolished immunoprecipitation of FLAG-SDCCAG8, demonstrating specificity of the interaction.

We next proceeded to validate SDCCAG8 interaction of the highest ranking RAB effector proteins, RABEP2, ERC1, and the centrosomal satellite protein CEP131. We used an SDCCAG8 co-immunoprecipitation assay followed by Western blotting in HEK293 kidney epithelial cells. We found that a transiently transfected FLAG-tagged full-length SDCCAG8 construct co-immunoprecipitated with endogenously expressed RABEP2, ERC1 and CEP131 proteins (**Fig 3B–3D**). In contrast, truncating fragments of SDCCAG8 corresponding to N-terminal, C-terminal and middle portion of the SDCCAG8 protein failed to immunoprecipitate RABEP2, ERC1 and CEP131, except for the C-terminal SDCCAG8 fragment that showed weak binding to RABEP2 (**Fig 3BiD**). We also performed a reciprocal co-immunoprecipitation experiment in FLAG-SDCCAG8 overexpressing control and siCEP131 knockdown cell lysates. While antibodies against CEP131 co-immunoprecipitated FLAG-SDCCAG8 from control cell lysates, non-specific anti-IgG antibodies failed to do so (**Fig 3E**). Similarly, antibodies against CEP131 did not co-immunoprecipitate FLAG-SDCCAG8 from siCEP131 knockdown cell lysates confirming the specificity of the interaction (**Fig 3E**). Since each of the SDCCAG8 interacting proteins contain multiple coiled-coil domains [30–32] it is likely that their interaction with SDCCAG8 is mediated through these domains, as we have demonstrated for SDCCAG8 interaction with OFD1 [1]. Together our data demonstrate that SDCCAG8 interacts with a distinct set of proteins including centriolar satellite components, endosomal vesicle components, tRNA synthesis components, and myosin type II motor proteins at the basal body.

### RABEP2, ERC1, and CEP131 localize to the cilia basal body

**Fig 4:**
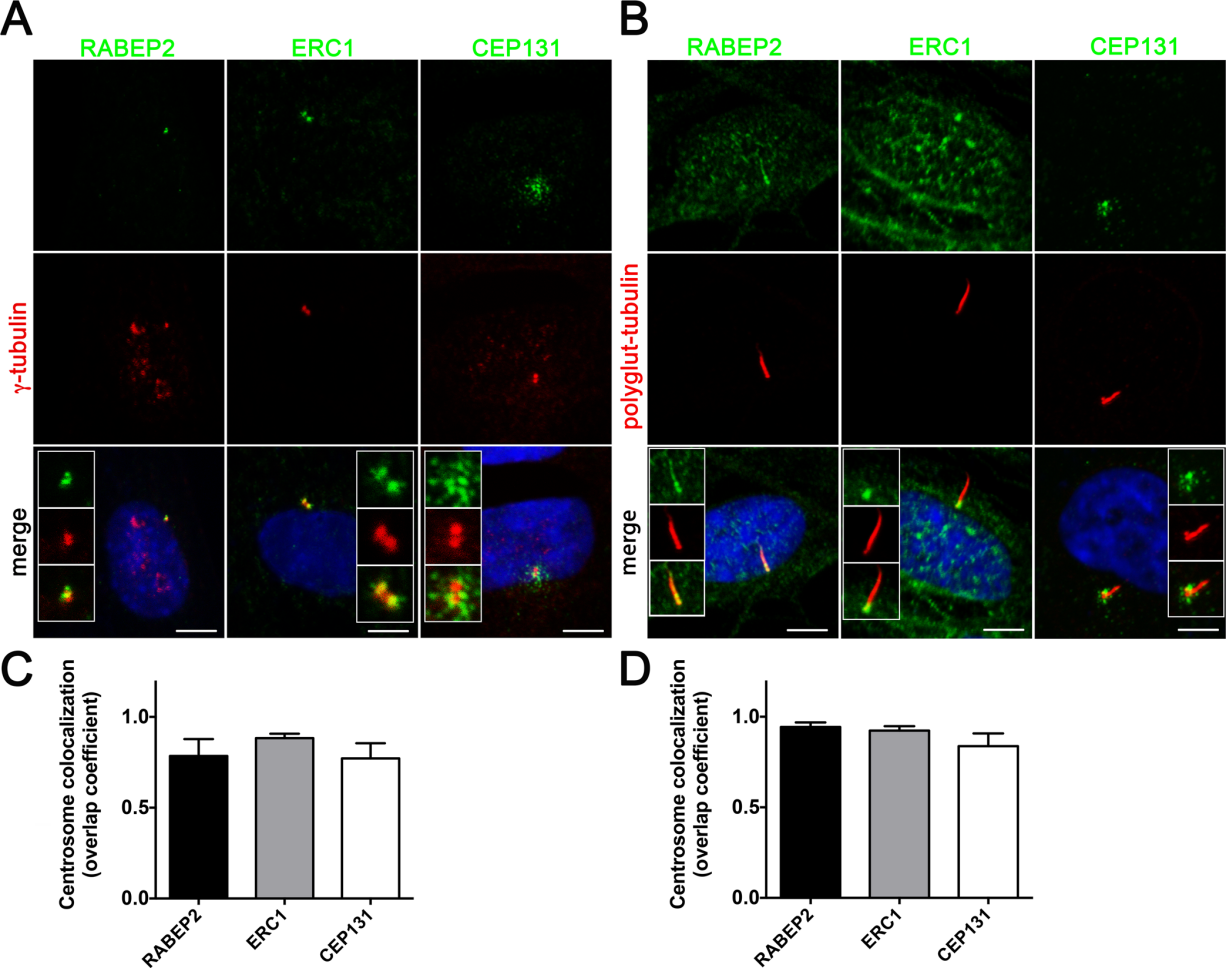
Proteins identified by SILAC assay as SDCCAG8 interactors localize to the basal body or centrosome. (**A**) Co-staining with the centriolar protein γ-tubulin demonstrates centrosomal localization for the identified SDCCAG8 interacting proteins RABEP2, ERC1, and CEP131 in hTERT-RPE1 cells. Note CEP131 localization also at centriolar satellites. Scale bars: 5 μm. (**B**) Co-staining with the ciliary axoneme marker poly-glutamylated tubulin (red) demonstrates ciliary and basal body localization of RABEP2, basal body localization of ERC1 and centriolar satellite localization of CEP131 in hTERT-RPE1 cells. Scale bars: 5 μm. (**C**) Quantification of the co-localization coefficients of RABEP2, ERC1 and CEP131 with γ-tubulin positive centrosomes in cells from (**A**). In each case n>40 centrosomes; error bars, SEM. (**D**) Quantification of the co-localization coefficients of RABEP2, ERC1 and CEP131 with polyglutamine-tubulin positive centrosomes in cells from (**B**). In each case n>40 centrosomes; error bars, SEM.

Since RABEP2 and ERC1 have not previously been reported as components of the cilium, we studied their intracellular localization using immunofluorescence analysis in hTERT-RPE1 cells. Non-ciliated hTERT-RPE1 cells were stained with antibodies against the centriolar protein γ-tubulin and each of the protein being examined (**Fig 4A**). RABEP2 and ERC1 localize to the centrioles of non-ciliated cells, while CEP131 is associated with the centriolar satellites, similar to previously reported data [12]. Staining of ciliated hTERT-RPE1 cells with antibodies against poly-glutamylated tubulin that labels the ciliary axoneme and each of the proteins, revealed RABEP2 localization both to the basal body and to the ciliary compartment (**Fig 4B**). ERC1 expression was retained at the basal body of ciliated cells, and CEP131 localized to the centriolar satellites (**Fig 4B**). To verify the centrosomal localization of RABEP2, ERC1 and CEP131 we quantitated the centrosomal colocalization coefficiencies of these proteins with γ-tubulin in non-ciliated cells, and with polyglutamylated tubulin in ciliated cells. Analysis of over 40 centrosomes in each condition revealed in average 78% of RABEP2, 85% of ERC1 and 77% of CEP131 colocalization with γ-tubulin in non-ciliated cells (**Fig 4C**), and 94% of RABEP2, 92% ERC1 and 84% of CEP131 colocalization with polyglutamylated tubulin in ciliated cells (**Fig 4D**). Together, our analysis demonstrates that RABEP2, ERC1 and CEP131 localize to the centrosome in hTERT-RPE1 cells. This is the first report to demonstrate centriolar localization for RABEP2 and ERC1.

### RABEP2 is required for ciliogenesis

**Fig 5:**
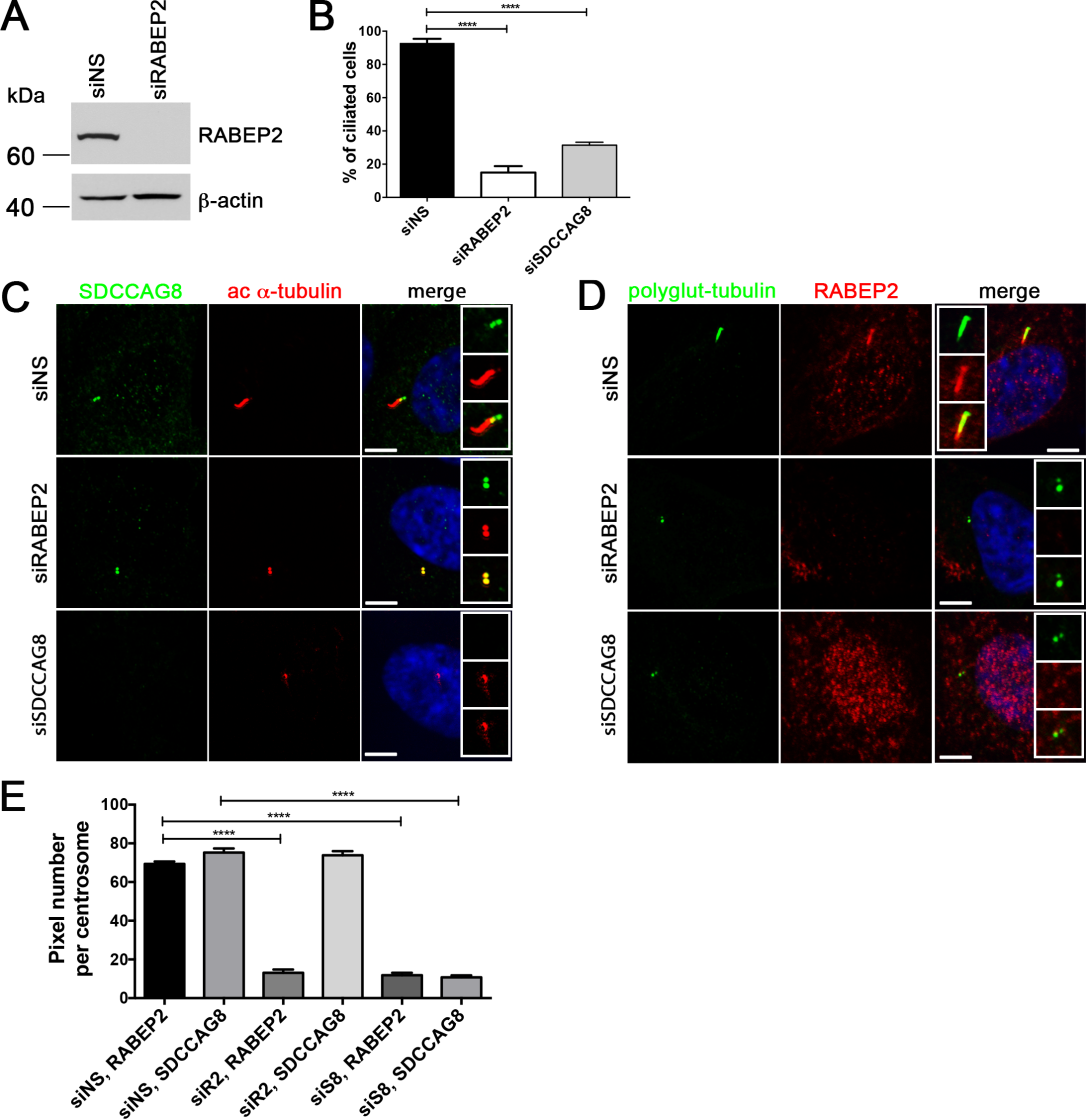
RABEP2 siRNA knockdown abolishes cilia formation. (A) Western blot analysis of extracts from control or siRABEP2-depleted hTERT-RPE1 cells, probed with anti-RABEP2 or anti-β-actin, as a loading control. Dharmacon SMARTpool siRNAs were used to knock down endogenous RABEP2 at high efficiency. (B) RABEP2 knockdown abolishes cilia formation in hTERT-RPE1 cells. Control, RABEP2 and SDCCAG8 depleted hTERT-RPE1 cells were serum starved for 48 hours after transfection with siRNA, fixed, and immunostained for acetylated α-tubulin to label primary cilia. Percentage of ciliated cells was determined in each case, control 92.8 ± 5.2%, n = 50; siRABEP2 15 ± 8.6%, n = 50 and siSDCCAG8 31.48 ± 1.694%, n = 50. Error bars, SEM, ****p<0.0001. (C) hTERT-RPE1 cells were transfected with non-specific siRNA (siNS), siRABEP2, or siSDCCAG8. Cells were ciliated by serum starvation and stained with antibodies against SDCCAG8 (green) and acetylated α-tubulin (red). Knockdown of RABEP2 abolishes cilia formation, but does not alter SDCCAG8 localization at the centrioles. Knockdown of SDCCAG8 abolishes cilia formation in hTERT-RPE1 cells. Scale bars: 5 **μ**m. (D) hTERT-RPE1 cells were transfected with non-specific siRNA (siNS), siRABEP2, or siSDCCAG8. Cells were ciliated by serum starvation and stained with antibodies against RABEP2 (red) and polyglutamylated tubulin (green). Knockdown of RABEP2 abolishes cilia formation. Knockdown of SDCCAG8 abolishes cilia formation and depletes RABEP2 localization from the centrioles in hTERT-RPE1 cells. Scale bars: 5 **μ**m. (E) RABEP2 localization at the centrosome is dependent on SDCCAG8. RABEP2- and SDCCAG8-positive pixels were quantitated at acetylated α-tubulin or polyglutamylated-tubulin positive centrosomes in siNS, siRABEP2 and siSDCCAG8 cells (C and D). While control cells (siNS) have 69.40 ± 1.194, n = 30, RABEP2-positive pixels at the centrosome, RABEP2-positive pixels are significantly reduced in siRABEP2 cells (13.00 ± 1.678, n = 30, ****p<0.0001) and in siSDCCAG8 cells (11.84 ± 1.231, n = 30, ****p<0.0001). Control cells (siNS) have 75.25 ± 2.145, n = 30 SDCCAG8-positive pixels at the centrosome, which is not changed in siRABEP2 cells (73.83 ± 2.138, n = 30), but is significantly reduced in siSDCCAG8 cells (10.76 ± 0.9907, n = 30, ****p<0.0001). Error bars, SEM; siNS, non-specific siRNA; siR2, RABEP2 siRNA; siS8, SDCCAG8 siRNA.

We demonstrated above that depletion of *Sdccag8* leads to loss of cilia in mouse embryonic fibroblasts and its knockdown in hTERT-RPE1 cells has a similar, albeit slightly milder effect on ciliogenesis. We next investigated whether a knockdown of RABEP2, the second most highly represented protein in our SILAC analysis has a cilia phenotype in hTERT-RPE1 cells. Effective depletion of RABEP2 was verified by Western blot and immunofluorescence of siRNA-transfected cells (**Fig 5A**). Depletion of RABEP2 or SDCCAG8 significantly (p<0.0001) reduced cilium formation in serum-starved cells, with only 15 ± 8.6% of RABEP2 knockdown, and 31.48 ± 1.694% of SDCCAG8 knockdown cells having a cilium, compared to 92.8 ± 5.2% wild type cells (**Fig 5B**).

Since, SDCCAG8 and RABEP2 proteins interact with each other, and the knockdown of either gene led to a similar effect on ciliogenesis in hTERT-RPE1 cells, we next asked whether their centrosomal co-localization is dependent on the presence of their interaction partner—SDCCAG8 or RABEP2, respectively. To address this question we used immunofluorescence staining against either of the endogenous proteins in hTERT-RPE1 cells, while the expression of its interacting protein was silenced by siRNA (**Fig 5C and 5D**). Although, knockdown of RABEP2 abolished cilia formation, SDCCAG8 protein was still localized to the centrioles in RABEP2 depleted cells, while SDCCAG8 was undetectable in SDCCAG8 knockdown cells (**Fig 5C**). In contrast, knockdown of SDCCAG8 lead to complete absence of RABEP2 from the centrosome in cells with the strongest knockdown effect of SDCCAG8, as judged by the absence of cilia formation (**Fig 5D**). To quantitate the effect of RABEP2 or SDCCAG8 knockdown on either protein’s centrosomal localization, we measured the number of RABEP2- and SDCCAG8-positive pixels at the centrosome (**Fig 5E**). There was a significant reduction in the amount of RABEP2-positive pixels at the centrosome in SDCCAG8 depleted cells, while the number of SDCCAG8-positive pixels remained unchanged by RABEP2 knockdown (**Fig 5E**). Thus, our data demonstrates that SDCCAG8 is required for RABEP2 centrosomal localization, while the localization of SDCCAG8 is independent of RABEP2.

**Fig 6:**
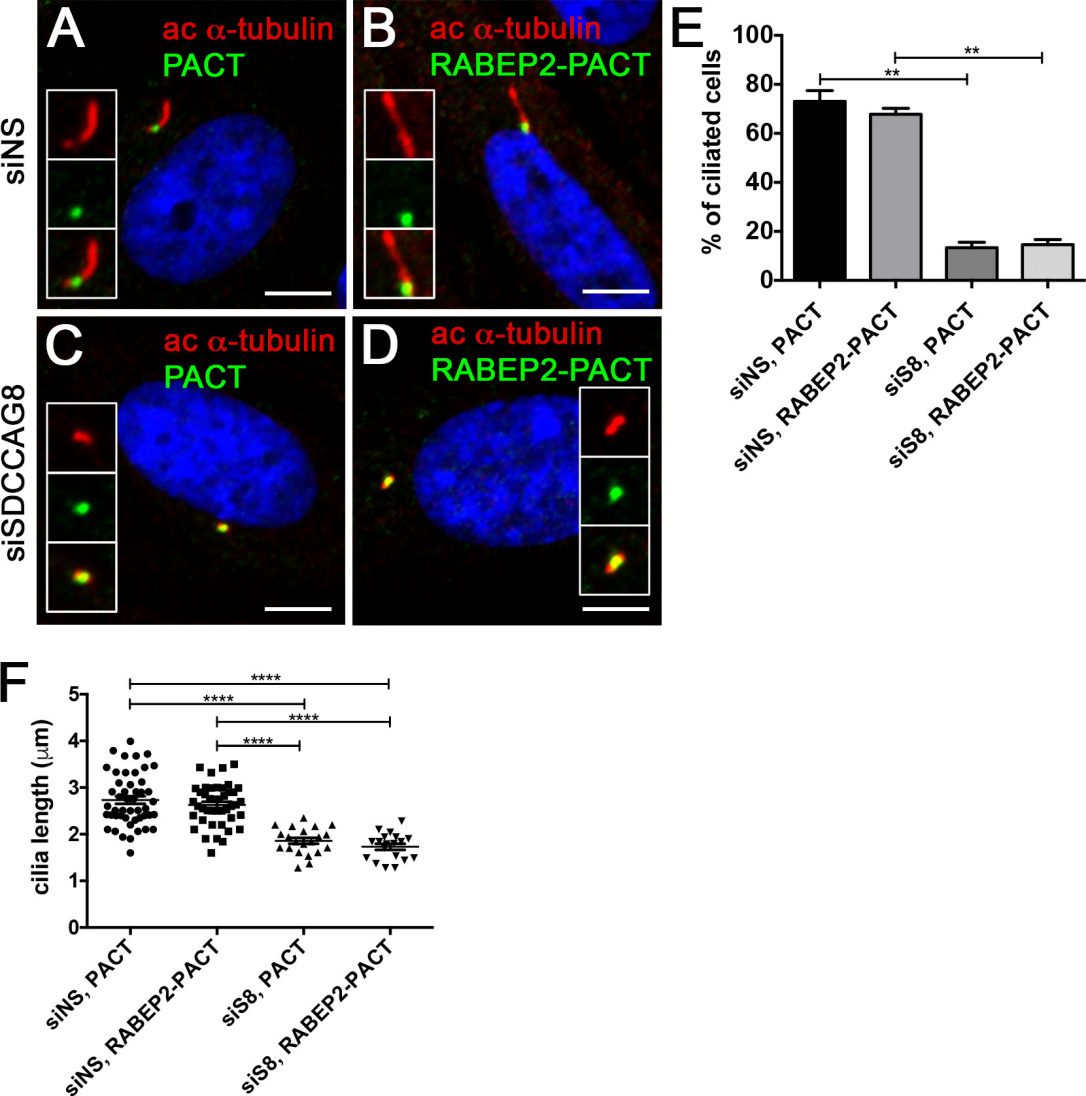
RABEP2-PACT does not rescue siSDCCAG8 ciliogenesis defect. (A,B) hTERT-RPE1 cells were transfected with non-specific siRNA (siNS) and 24 hours later transfected with EGFP-PACT (A) or EGFP-RABEP2-PACT (B) constructs. Either of the tagged proteins localized to the centrosome and were detected with anti-GFP antibody (green). Ciliogenesis (acetylated-α-tubulin, red) was not affected in either of the situations. (C,D) hTERT-RPE1 cells were transfected with siSDCCAG8 and 24 hours later transfected with EGFP-PACT (C) or EGFP-RABEP2-PACT (D) constructs. Although, the tagged proteins localized to the centrosome (green), they failed to rescue the ciliogenesis defect (acetylated α-tubulin, red) in *SDCCAG8* knockdown cells. Scale bars: 4 **μ**m. (E) Quantitation of the percentage of ciliated cells in (A, B, C and D), demonstrates that over expression of the EGFP-PACT or EGFP-RABEP2-PACT construct does not affect normal ciliation in siNS hTERT-RPE1 cells. Moreover, EGFP-RABEP2-PACT construct fails to rescue the ciliation defect in SDCCAG8 depleted cells (**p = 0.0068). Error bars, SEM. (F) We measured the length of cilia in EGFP-PACT and EGFP-RABEP2-PACT overexpression cells to examine whether it was affected. There was no significant difference in the length of cilia between siNS cells expressing EGFP-PACT and EGFP-RABEP2-PACT (73.08 ± 4.417 **μ**m, n = 3, vs. 67.80 ± 2.446 **μ**m, n = 3, p = 0.41). Similarly, the cilia length was uncorrected by the overexpression of EGFP-PACT and EGFP-RABEP2-PACT in siSDCCAG8 cells (13.36 ± 2.194 **μ**m, n = 3 vs. 14.58 ± 2.083 **μ**m, n = 3, p = 0.72). Error bars, SEM.

Since we have shown that RABEP2 is essential for ciliogenesis and its localization at the centrosome is abolished in SDCCAG8 knockdown cells, we next asked whether RABEP2 functions downstream of SDCCAG8 in ciliogenesis. To address this, we examined whether tethering RABEP2 to the centrosome would rescue the ciliogenesis phenotype in SDCCAG8 knockdown cells. We expressed EGFP-PACT or EGFP-RABEP2-PACT constructs in hTERT-RPE1 cells that were prior transfected with either siNS or siSDCCAG8 (**Fig 6**). Both over expressed fusion proteins localized to the centrosome and had no effect on ciliogenesis in hTERT-RPE1 cells transfected with non-specific siRNA (**Fig 6A, 6B, 6E and 6F**). As expected, over expression of EGFP-PACT in SDCCAG8 knockdown cells did not rescue the ciliogenesis defect (**Fig 6C, 6E and 6F**). Interestingly, also EGFP-RABEP2-PACT over expression failed to rescue the ciliogenesis defect in SDCCAG8 knockdown cells (**Fig 6D, 6E and 6F**). This result suggests, that 1) SDCCAG8 is indispensable for RABEP2 centrosomal/ciliary function, or 2) RABEP2 must be able to traffic into the cilium for its function in ciliogenesis. Unfortunately neither of the scenarios can be easily tested. Together, our results demonstrate that RABEP2 centrosomal localization is regulated by SDCCAG8, and its function is critical for ciliogenesis.

## Discussion

Although mutations in *SDCCAG8* were demonstrated to cause a retinal-renal ciliopathy in association with Senior-Loken syndrome and BBS-like features, the function of SDCCAG8 within the cilium and its associated structures n of SDCCAG8 within the cilium and its associated structures–centriolar satellites and basal body is poorly understood [1]. We previously reported the non-ciliary functions of SDCCAG8 in the regulation of DNA damage response signaling [3, 33]. In this study we have examined the cilium-related function of SDCCAG8 during mouse embryonic development and characterized its protein interaction complex at the basal body using a SILAC proteomics assay combined with immunofluorescence (IF) analysis. We find that SDCCAG8 associates with RAB effectors and other vesicle transport components at the base of the cilium. In addition, we show that the SDCCAG8 interacting protein RABEP2 regulates ciliogenesis.

### *Sdccag8* and Hedgehog Signaling Defects

In this study we report, that *Sdccag8*^*gt/gt*^ mice develop skeletal malformations of rib cage, pre-axial polydactyly and triphalangeal thumbs, features that are characteristic of various ciliopathies, such as Meckel-Gruber syndrome, Jeune Syndrome and Bardet-Biedl syndrome in humans [34]. However, polydactyly in BBS is associated with post-axial digital malformations and none of the BBS mouse models characterized to date, present this feature [35–38]. Sternal ossification defects and formation of pre-axial polydactylous triphalangeal thumbs preferentially in the hind limbs have been described in two different mouse models of Meckel-Gruber syndrome gene *Mks1*—*kerouac* and *Mks*^*del64-323*^ and demonstrated to be caused by disruptions in Hh signaling [39, 40]. To functionally investigate the ability of *Sdccag8*-depleted cells to activate Hh signaling we used *Sdccag8*^*gt/gt*^ derived MEFs. Our analysis showed that the mutant cells have an attenuated response to Hh pathway activator SAG, compared to wild type cells. As an alternative model to study Shh activity we investigated Sonic hedgehog dependent neural tube patterning along the dorsal-ventral axis. Since neural progenitor specification is dependent on the dose and duration of Shh activity on each neuron subtype this model allows for sensitive detection of changes in Shh pathway activity. However, our analysis failed to detect a Shh pathway defect in this tissue, indicating that Sdccag8 is not essential for neural tube patterning.

Hh signaling in mammals is dependent on intact cilia [5]. Although, our proteomics assay did not reveal SDCCAG8 protein interaction with Hh signaling pathway components the observed Hh signaling defects may be caused by a more general ciliogenesis defect in *Sdccag8*^*gt/gt*^ animals. In contrast to our previous report in the kidney, we found that abrogation of *SDCCAG8* significantly impaired cilia growth in *Sdccga8*^*gt/gt*^ derived MEFs in cell culture [3]. These findings were also recapitulated in human hTERT-RPE1 siSDCCAG8 knockdown cells, where partial knockdown of SDCCAG8 caused a significant shortening of the length of the cilium. It is intriguing however, that despite the dramatic ciliary and Shh phenotypes in *in vitro* cell culture models, the *in vivo* role of Sdccag8 in modulating Hh signaling appears to be restricted to a few tissues—the developing rib cage and limb buds, as our analysis of *Sdccag8*^*gt/gt*^ mice did not reveal any other gross abnormalities that are frequently associated with impaired Hh signaling, such as exencephaly, hydrocephalus or cleft palate. Furthermore, the great variability in the penetrance of the polydactyly phenotype suggests that Sdccag8 does not directly interact with Hh signaling pathway components, but rather has an indirect effect (i.e. through regulating cilia growth or function).

### SDCCAG8 and ciliogenesis

Cilia growth is dependent on simultaneous axonemal elongation and membrane extension at the basal body. Our immunofluorescence analysis suggests that the first stages of ciliogenesis, including basal body maturation (defined by the presence of CEP164, CEP83 and FBF1-positive distal appendages), are unaffected in *Sdccag8*^*gt/gt*^ cells [24, 41]. However, subsequent growth of the cilium was impaired, suggesting a requirement for SDCCAG8 function in some aspect of this process. By combining affinity proteomics with biochemical and IF analysis we find that SDCCAG8 regulates intracellular membrane/vesicular trafficking to the base of the cilium. Although the precise mechanism remains to be studied, we speculate that SDCCAG8 may function as an adapter that links cargo proteins to actin based motors (i.e. MYH9, MYH10 or MYH14) through its interaction with membrane associated RAB effectors (RABEP2, ERC1, ERC2). Once the vesicles have reached the centrosome SDCCAG8 may be involved in their docking to centriolar satellites through binding to OFD1 and CEP131 [1]. Involvement of SDCCAG8 in membrane trafficking would also provide a functional explanation for the retinal outer segment degeneration phenotype in *Sdccag8*^*gt/gt*^ mice, where we have shown a severe rhodopsin transport defect in the photoreceptor cells [3]. Since photoreceptor outer segments (equivalent of the cilium) have extremely high membrane turn over rates, any interference of membrane delivery to this structure is expected to have dramatic effect on outer segment maintenance [42].

Identification of SDCCAG8 association with CEP131 is intriguing, since CEP131 was recently demonstrated to interact with BBSome complex and regulate its ciliary targeting, and given the high phenotypic overlap between individuals with mutations in *SDCCAG8* and those with mutation in BBS genes [13]. However, it should be noted that we did not recover any other BBSome complex proteins in our affinity proteomics assay and SDCCAG8 has not been reported to interact with BBSome, suggesting that the functions of SDCCAG8 and BBSome are only partially overlapping [8, 13, 26].

An exciting finding from the SILAC assay was the identification of several RAB effectors as novel interaction partners of SDCCAG8, none of which had been previously associated with the cilium. We demonstrated enrichment of RABEP2 and ERC1 at the centrosome, and localization of RABEP2 within the cilium by IF. RABEP2 (previously named Rabaptin-5beta, due to 42% identity to Rababtin-5) was originally identified by yeast two-hybrid screen as an effector of RAB5. The protein was shown to interact with the RAB5 exchange factor Rabex-5 and regulate RAB5 mediated endosomal fusion [30]. It contains 3 coiled-coil domains and a C-terminal RAB-binding domain. This work discovered RABEP2domcritical role in ciliogenesis by using siRNA knockdown. Our data suggest that RABEP2 localization at the centrosome is regulated by SDCCAG8, since knockdown of SDCCAG8 abolished RABEP2 centrosomal localization. We also demonstrated that RABEP2 is a component of the cilium, suggesting that it may be a component of the intraciliary transport mechanism. Further supporting a role of RABEP2 in the cilium is our demonstration that centrosomally targeted RABEP2 failed to rescue the ciliogenesis defect in *SDCCAG8* knockdown cells. However, more work is needed to uncover the precise mechanism of RABEP2 recruitment at the centrosome and its function within the cilium.

ERC1 (also known as RAB6IP2 or ELKS) has been previously shown to be a component of the NF-κB signaling cascade, of the synaptic vesicles at the active zone of synapses, and implicated in RAB6 mediated excretory pathway in pancreatic β-cells [31, 43–45]. Similarly to RABEP2 function, ERC1 is required for proper vesicle docking and fusion at the membrane [46]. In its absence RAB6- and RAB8-positive vesicle docking was mistargeted and fusion to cell membrane delayed [46]. Although, homozygous *Erc1*^*-/*-^ mice have been generated, unfortunately they are embryonic lethal, and their phenotype has not been characterized [44].

SDCCAG8, a subdistal appendage component [1], joins the list of ciliary proteins, which regulate cilia growth through promoting vesicle docking to the basal body. Identification of its interaction partners RABEP2, ERC1 and ERC2, none of which have been previously implicated in ciliogenesis, demonstrates further the complex regulation of ciliogenesis and expands the already recognized functions of these proteins (synaptic vesicle trafficking, exocytosis, NFb signaling). Further studies are needed to understand how these RAB effector proteins interact with other RAB cascades characterized in ciliogenesis (i.e, RAB8, RAB11, Rabin8, Rabep1) [8, 14–17].

## Supporting Information

S1 Fig Sdccag8 function is not required for neural tube patterning. (**A**–**H**) Immunofluorescence images of sections through E10.5 wild type (**A**,**C**,**E**,**G**) and *Sdccag8*^*gt/gt*^ (**B**,**D**,**F**,**H**) neural tubes at the level of the hindlimb. (**A**,**B**) FoxA2 marks floor plate cells, (**C**,**D**) Hb9 marks motor neurons, (**E**,**F**) Nkx2.2 marks V3-interneuron progenitors, and (**G**,**H**) Nkx6.1 labels V2-, V3- and motor neuron progenitors. No changes in the expression domains of the different neuronal markers were observed in *Sdccag8*^*gt/gt*^ vs. wild type embryos. Scale bar: (**A**–**H**) 50 μm.
(TIF)
