# Supplementary figures and images for: SDCCAG8 Interacts with RAB Effector Proteins RABEP2 and ERC1 and Is Required for Hedgehog Signaling
Source: PLoS One. 2016 May 25;11(5):e0156081. doi: 10.1371/journal.pone.0156081 (PMC4880186; doi:10.1371/journal.pone.0156081)

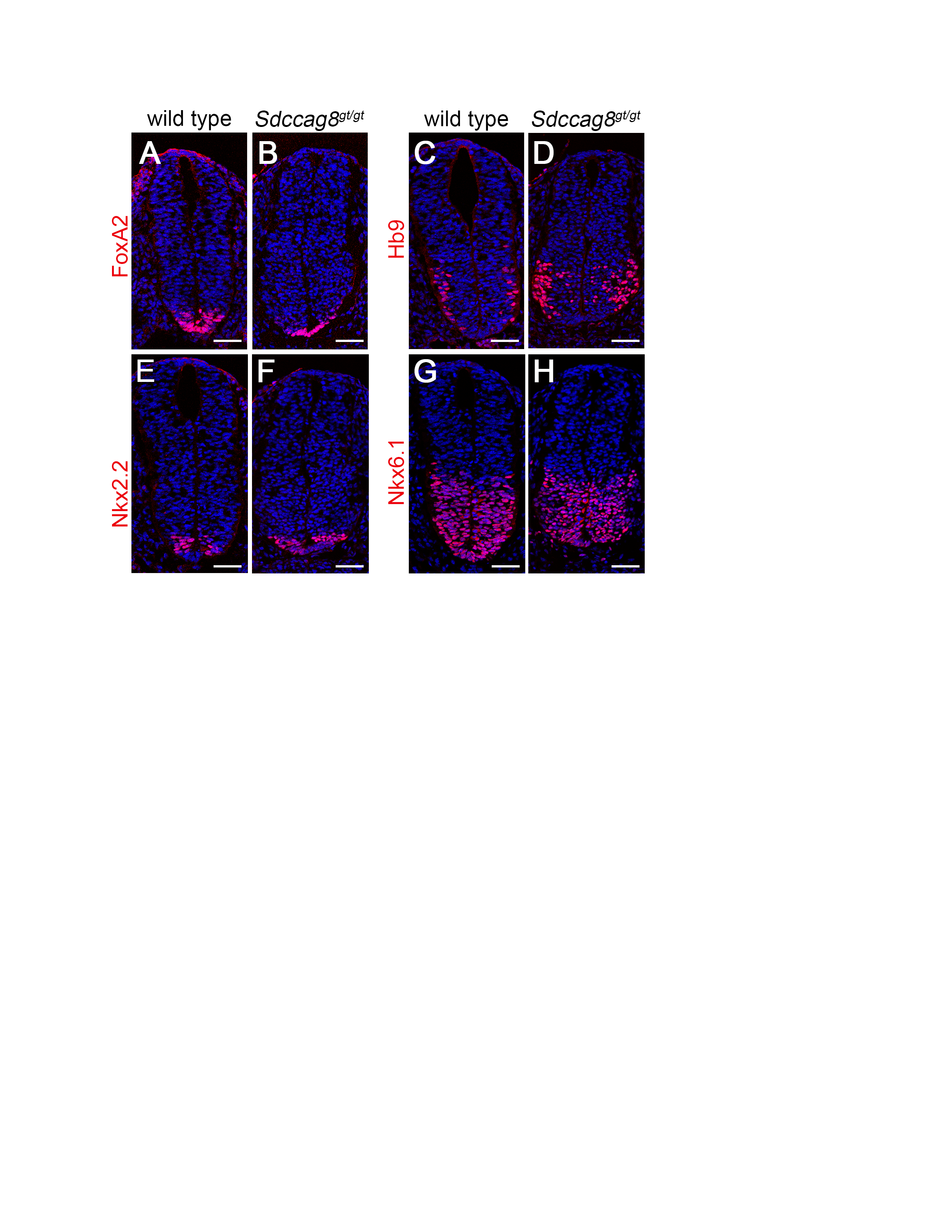

Supplement: S1 Fig — (A–H) Immunofluorescence images of sections through E10.5 wild type (A,C,E,G) and Sdccag8gt/gt (B,D,F,H) neural tubes at the level of the hindlimb. (A,B) FoxA2 marks floor plate cells, (C,D) Hb9 marks motor neurons, (E,F) Nkx2.2 marks V3-interneuron progenitors, and (G,H) Nkx6.1 labels V2-, V3- and motor neuron progenitors. No changes in the expression domains of the different neuronal markers were observed in Sdccag8gt/gt vs. wild type embryos. Scale bar: (A–H) 50 μm. (TIF) [file pone.0156081.s001.tif]
